# One Health: A social science discussion of a global agenda

**DOI:** 10.1051/parasite/2022014

**Published:** 2022-03-22

**Authors:** Jean Estebanez, Pascal Boireau

**Affiliations:** 1 Université Paris Est Créteil, Lab’Urba 77420 Champs-sur-Marne France; 2 ANSES, Laboratoire de santé animale 94700 Maisons-Alfort France

**Keywords:** One Health, Social sciences, Biodiversity, Capital, Local knowledge, Non-human knowledge

## Abstract

This article introduces the *Parasite* issue dedicated to part of the research in social sciences supported by the *Domaine d’Intérêt Majeur de la Région Île-de-France* (DIM) One Health [2016–2022]. We show how the four papers of this special issue are related. Jérôme Michalon recalls the genealogy of One Health and analyzes it as an “epistemic watchword”. Using antibiotic resistance as a case study, Estera Badau demonstrates how “One Health” results from a series of formulas and the bringing together of a plurality of fields and actors. Nicolas Lainé and Serge Morand show how One Health fits in with attempts already initiated in the colonial period and context. They highlight the need to (re)legitimize local and non-human knowledge, in order to truly decolonize One Health and better prevent epidemic emergence. Finally, Frédéric Keck, Nicolas Lainé, Arnaud Morvan and Sandrine Ruhlmann show how zoonotic reservoir and cultural practices are linked in the context of three specific societies. This paper highlights two main contributions of social sciences: 1) To think about One Health genealogy, how the question is framed and by which actors. The questions of practices, social representations but also of the environment are less present than the issues of human and animal medicine. The Anthropocene, the Capitalocene, even some of its variations such as the “domesticoscene” thus appear to be key elements. 2) To propose methods and tools that make One Health operational, advocating a less asymmetrical view of types of knowledge (scientific, local, non-human) and more contextualized global health recommendations.

## Abstract (fr)

Cet article introduit le numéro spécial de la revue *Parasite* dédié à des travaux soutenus par le Domaine d’Intérêt Majeur de la Région Île-de-France *One Health* [2016–2022]. Nous montrons ainsi l’articulation entre les quatre articles constituant ce numéro. Jérôme Michalon rappelle la généalogie de *One Health* et propose une définition qui engage : un mot d’ordre épistémique. Estera Badau illustre le propos avec le cas de l’antibiorésistance en montrant comment *One Health* résulte d’une série de formulations et de la mise en relation d’une pluralité de domaines et d’acteurs, notamment scientifiques, gestionnaires ou porteurs de politiques publiques. Nicolas Lainé et Serge Morand montrent la façon dont *One Health* s’inscrit dans des tentatives déjà initiées en période et contexte colonial. Dans ce cadre, ils mettent en avant la nécessité de (re)légitimer le savoir local et celui des non-humains, afin de réellement décoloniser *One Health* et de mieux prévenir les émergences épidémiques. Enfin, Frédéric Keck, Nicolas Lainé, Arnaud Morvan et Sandrine Ruhlmann montrent comment réservoir zoonotiques et pratiques culturelles s’articulent dans le cadre de trois sociétés spécifiques. Cet article met ensuite en avant deux apports principaux des sciences sociales. 1) réfléchir à la généalogie de *One Health*, à la façon dont la question est cadrée et par quels acteurs. Il apparait ainsi que les questions de pratiques, de représentations sociales mais aussi d’environnements sont moins présentes que les enjeux de médecine humaine et animale, alors même que les zoonoses sont fortement liées à des modifications des relations entre humains, animaux et environnement. L’anthropocène, et sans-doute plutôt le capitalocène, voire certaines de ses déclinaisons, comme le « domesticoscène » apparaissent ainsi comme des éléments clés. 2) Proposer des méthodes et des outils qui permettent de rendre opérationnel *One Health*, en plaidant pour une vision moins asymétrique des types de savoirs (scientifiques, locaux, non-humains) et plus contextualisée des recommandations de santé globale.

This special edition of the *Parasite* journal will present research that is supported, developed and, more generally, belongs to the scientific field from which the *Domaine d’Intérêt Majeur de la Région Île-de-France* (DIM) One Health (www.dim1health.com) has emerged [2016–2022]. The DIM is founded on an approach that brings together animal, human, and environmental health [2, 3], and its activity is anchored in a multi-disciplinary approach to infectiology that includes monitoring, diagnostics, microbiology, parasitology, therapy, vaccinology, immunology, anthropology, sociology, geography and communication. The DIM is active in the implementation of One Health (OH) thanks to its organisational and financing activities, and aims to support research through a reflective approach. Contributions from the fields of social science have been included here in a journal traditionally concerned with life sciences, so that they may participate in the multidisciplinary discourse on the notion of OH [6, 17].

The ongoing COVID-19 pandemic has placed bats at the heart of global health. Alongside rodents and influenza in the Middle Ages, and the bird flu of the 19th century, chiropterans [9] have been designated a major reservoir of virus pandemics. Although their specific role in the emergence and spread of COVID-19 is a matter of debate, the now-crucial question of zoonotic transmission has prompted the widening of our parameters of health to include animals, plants and the environment. Global health thus appeared in the 1990s as a global movement which initially questioned the functioning of international organisations such as the WHO and aimed at greater globalisation and a greater thematic openness. Thus, global health takes the form of a range of mechanisms that assist us in preparing for, detecting, and limiting future epidemics, following the approach advanced by OH.

As J. Michalon [14] lays out in this edition, thanks to a tripartite collaboration between the World Health Organization (WHO), the World Organisation for Animal Health (OIE), and the United Nations Food and Agriculture Organisation (FAO), the One Health approach, updated in 2008, has unsurprisingly been advanced by its promoters as a crucial point of reconciliation between human and veterinary medicine, for the purpose of analysing the emergence and circulation of infectious diseases between species, and their ties to the environment (for this critical evolution, see the articles of J. Michalon [14] as well as N. Lainé and S. Morand [10] in this special edition). This milestone is generally associated with a series of world health crises at the end of the 1990s, including mad cow disease, genetically modified plants with a gene that is resistant to a specific antibiotic, and the spread of the HIV epidemic, as they led to the structuring of risk analysis and to the introduction of a protocol for the precautionary principle. By exposing the organisational and scientific limitations of the systems employed by the actors responsible for the management of questions of world health, the SARS virus, and to an even greater extent the H5N1 virus, led to the emergence of OH as a way of overcoming new threats through its systemic and holistic approach. The broad character of the initiative, driven by shared values such as sustainability, equality, modernity, and the quest for greater knowledge, also facilitated its adoption, while still allowing research into interests that were partially conflicting between institutions. Therefore, OH had the appearance of a new tool that helped to mitigate inter-organisational tensions, while leaving the question of the operational agenda partially unresolved.

Conversely, a second conversation in OH, led principally by veterinarians, has stressed the antiquity of this awareness of the ties between human, animal, and environmental health. This ecological view has only been maintained in veterinary medicine, which covers many animal species, whereas human medicine has become extremely specialised. The term OH is therefore no more than a formalisation and extension of the practices that have always been conducted by veterinarians, and consequently, they should be given a prominent place in any programmes that are to be developed.

These apparently contradictory accounts from actors are commonly used as instruments of power by which the issue can be contextualised, to legitimise the position of those who have constructed them. A. Cassidy [5] shows that the evolution of OH since the 1950s has been brought about through multiple attempts, many of which were unsuccessful, to promote its integrative qualities. Its emergence in the 21st century is a consequence of a coming together of two schools of thought: the connections between human and animal health previously developed within the two contexts of One Medicine, and One World One Health.

One Medicine, which is associated with the veterinarian and epidemiologist Calvin Schwabe, belongs in the continuity of comparative medicine and the development of veterinary public health, and for the most part engages scientific actors who are interested in epistemic questions. N. Lainé and S. Morand [10] show that these practices have developed within a long history that has its origins in the colonial period. One World One Health has closer associations with the field of international relations, in particular with non-governmental organisations. It uses an applied and targeted approach to science, in the service of public policy and public health managers. OH can thus be seen as a meeting point between the practice of academic research and a more institutional approach to action. This coming together marks a new direction – from scientific knowledge where the intrinsic legitimacy is sufficient, to knowledge that is put to the service of social goals, and is perhaps even shaped by the questions raised primarily by the actors themselves, who are outside the scientific world but are actors in public health, industry, or state.

E. Badau’s article [1] illustrates this with the case of antibiotic resistance – often presented as the paradigm of the OH approach – which allows a plurality of simultaneous actions to potentiate thanks to global governance. The article focuses on those sectors, including public health, animal health, aquaculture, agriculture and food supply, where anti-infectives have been used inappropriately, setting the stage for future crises. The question of antibiotic resistance has led to the progressive development of relations between different fields, including food safety, hospital treatment and health management, livestock farming practices and even the treatment of plants. Nevertheless, it seems that significant differences remain between the level of scientific knowledge, and political decision-making, on how to respond to the crisis and the shortage of anti-infectives, which are becoming a rare commodity and need to be employed with the utmost caution, and overseen by major planning and training for health workers.

If the emergence of OH can be seen from the viewpoint of an institutional crisis resulting from a health crisis, it can also be considered as a proposition for institutional transformation, resulting in a spectacular coming-together of scientific institutions and international agencies, of research and public policy. OH actively promotes interdisciplinary cooperation (as can be seen in other fields [11]) which thus validates the position of some disciplines, such as veterinarian sciences, that have worked in this field.

J. Michalon [14] even proposes that OH is above all an “epistemic watchword”, which, in spite of its lack of precision, or perhaps because of it, has become effective by directing actors to work in partnership, in order to produce expert knowledge on the connections between human, animal and environmental health. This perspective builds on N. Lainé and S. Morand’s [10] account of OH’s emergence during the colonial period, due to the political and logistical challenges surrounding health, for example, treating humid zones to reduce malaria. We could also consider the declarations made by UNESCO during decolonisation, regarding the necessity of conserving biodiversity in a general sense. This epistemic watchword can also be found in the definitions of antibiotic resistance presented by E. Badau [1]. If antibiotic resistance and OH are evolving, there is also a tendency to differentiate themselves from each other. One of the objectives of health workers has been the mobilisation of society as well as a stimulant to the economic sector.

A primary contribution by social sciences is to lay out the evolution of the notion of OH, in a way that is not limited to accounts given by actors invested in presenting a specific vision, but which does not reject the factual and analytical elements these actors propose. This evolution, which opens this edition with J. Michalon’s article, is central because it allows us to better understand how the issue has been framed: What questions have been asked? By which actors? Coming from which disciplines? And conversely, which are the questions that are seldom asked, or never asked at all? Which are the disciplines whose ideas are less heard? How can OH be defined: is it an organisation, an agenda, or a watchword? It seems that although the social sciences are certainly not invisible in OH, as this edition proves, they do not occupy a central position despite an increasing number of calls for better integration. In the same way, the ecological sciences seem to be less visible when compared to infectiological and parasitological investigations and projects. It needs to be pointed out that very few of the projects submitted and supported by DIM1HEALTH over the past 5 years were really concerned with this discipline: out of more than 250 research projects submitted to DIM1HEALTH over 5 years of call to tender, only 5 proposals concerned environmental approaches, and of those, three received funding. The environmental dimension of OH therefore seems to be far less central than questions of human and animal health, not because the challenges faced are less important, but because ecologists and social science specialists are not (yet) central actors. This is undoubtedly one of the limitations of disciplinary openness, which does not only concern OH, but among many issues, conservation. Yet in his time, Pasteur himself started with fermentation, before moving to chicken cholera, and from there to human vaccination against rabies, all within the field of microbiology. N. Lainé and S. Morand [10] demonstrate how anthropology, the discipline most closely associated with the colonial period, is going to disappear from the field of health in the decades following its end. The recent reformulation of OH thus seeks to integrate the social sciences in this field, and it is anticipated that they will participate in the acceptability of prophylactic measures, defined by other disciplines, and that they will bring some objective insight into, and from, local knowledge. The social and human sciences are becoming more and more important because they explain the local risks from emerging infectious agents in the context of a changing host species: cultural practices, in particular funerals, were a major engine for the spread of the Ebola virus, and in the case of SARS-CoV-2, although its origin is still not known, the spread or increase of the earliest cases very certainly benefited from wet markets, where live animals are sold. Authors highlight the many ways the social sciences ‘allow the transformation of our knowledge’. In parallel, ethnobiology can interpret how reservoirs of infectious agents function, by repositioning the animals and humans in their environment. N. Lainé and S. Morand [10] illustrate how these human sciences can contribute to limiting recurrent anthropocentricism when speaking of living beings, because animals are not merely simple objects. In light of this, it makes increasing sense to bring together actors who are in permanent contact with domestic, captive and wild animals, as well as working towards a better understanding of the measures that are imposed locally in times of crisis and their acceptability. The role of social sciences is precisely to describe what people do locally. Indigenous knowledge, as well as animal knowledge, is becoming more and more vital in the face of a very limited chemical arsenal against infectious agents.

OH can be seen as a response to the ecological transformations humans have made to the world, both because of the long-term growth of connections between locations and populations during the course of globalisation, and because of the considerable impact their activity has had on their environment since the industrial revolution. This activity appears to be a real driving force for epidemics, thanks to the establishment of relations across different parts of the world, the expansion and intensification of the use of soils and the oceans, the consumption of resources, and global warming tied to the use of “machines” over time. Thanks to the widespread yet very unequal forming of relationships that characterises globalisation, the circulation of pathogens has reached new heights. Examples of this include the obliteration of 80% of the indigenous population of the Americas following the travels of Cristopher Columbus [15], pathogens (such as SARS-CoV-2) which have spread across the globe in a matter of weeks, wars [7] that have inspired the use of pathogens as weapons, the populating of areas that traditionally had few inhabitants and the creation of inter-species relations where none existed in the past. The emergence of the Anthropocene would appear to be central to OH, although it is in fact peripheral. This term was popularised by P. Crutzen at the beginning of the 21st century, allowed for a signalling of the end of the Holocene, which ushered in a new geological age brought about by human activity [12]. Many factors have been put forward to define the Anthropocene, including human sedentarisation and the path agriculture has taken over time in the recomposition of ties between living beings [4]. S. Morand [16] shows, for example, that the biomass of domestic animals today outweighs that of wild vertebrates by a factor of 100 (and this could lead us to rename the new era “Domesticocene” as a current geological age characterised by the dominant biomass of warm-blooded domestic vertebrates and their visible impact on biodiversity and the degradation of biotope), because of production models, the rapid increase of the human population, and the massive collapse of the biodiversity of vertebrates. Industrialisation has triggered other factors, from the recomposition of the relationship with natural resources and their limitless consumption, to the release of new particles into the atmosphere.

Some authors suggest that, more specifically than human activity in general, the origins of the major changes that we have witnessed over the past two generations lie fundamentally with the specific human activity of global capitalistic production [13], and thus we have entered the Capitalocene. The rationale of accumulating capital and machines (the one being tied to the other) and ever-increasing production, has driven a major loss of biodiversity through the destruction of habitats and enforced human, animal, and even plant migration because of global warming. Human activity has also led to homogenisation, and therefore weakening, of livestock species thanks to the demands of productivism, not to mention the immoderate use of antibiotics to the same end, which is causing the emergence of major antibiotic resistance. Thus, E. Badau’s [1] article demonstrates that this issue has become a major challenge for OH. These ecological changes seem to be a major driving force in the emergence of infectious agents, in particular of zoonotic agents. A range of transformations tied to human activity are effectively driving an intermingling of infectious agents on the world’s surface, a good example being the diffusion of the SARS-CoV-2. This is also driving impacts between pathogen reservoirs, domestic animals, human populations, and inducing microbiota and holobiont changes, which are leading to modifications in the virulence of infectious agents, and in the susceptibility of their hosts.

A second aspect of the relationship between social sciences and OH is that they do not only regard OH as a social object and analyse it accordingly, but rather participate in its implementation by promoting its capacity to actualise and understand human behaviour through a full range of specific techniques, including interviews, questionnaires, observations, and the use of statistics. Transforming individual practices into collective ones is central to the OH project: the ability to represent social relationships in the areas of health, disease, and animals, while also taking into account multiple inequalities is a valuable tool for promoting relevant transformations within the groups that are targeted and making these transformations effective. This model is sometimes limited in a very asymmetrical way, by the social acceptability of effecting the acceptance of recommendations that have been developed in another context, like for example norms that have been agreed on and imposed by backers from rich countries.

In a far more symmetrical model than this version, norms and recommendations can be built in collaboration with the groups effected, to engage them in what is being proposed, and make the pre-planned measures pertinent and effective. As proposed in N. Lainé and S. Morand’s [10] article, following the rationale of populations and of their relationship to animals is not necessarily the prelude to putting in place recommendations that will lead to a change in practices, as can be seen in the example of the analysis of the evolution of tuberculosis in Laotian pachyderms. Autochthon knowledge, even when developed by animals, such as auto-medication, can also lead to functional rationale, practices and types of relationships between humans, animals and the environment, that can call in to question the relevance of some public policy. In the same way, by taking the examples of the different rationales of three contrasting societies managing diseases and their circulation in Australia, Laos and Mongolia, F. Keck, N. Lainé, A. Morvan and S. Ruhlmann [8] show in their article how cultural practices can act as a mediation tool, and even control the infectious diseases emerging from animal reservoirs.

Although one must evidentially not fall into naive analyses that seem to aim at a better understanding of nature and thus be better adapted (this ontological distinction barely exists in the groups under consideration), it is also important to refrain from thinking of global health recommendations as necessarily the most effective in all contexts.

The question being posed here is the legitimacy of knowledge, not only in terms of the scientific disciplines included in OH, but also in the hierarchy of different types of knowhows and their integral capacity to be considered as full-fledged knowledge. Within the structure of OH, where there is a dual purpose of action and connection with public action, the invisibility of a large amount of local knowledge is evidently a political challenge, even as OH protocols (from Rio to Nagoya), demonstrate their importance in preserving the ecological quality of regions, as well as their biodiversity.
